# The effects of increasing dietary total Ca/total P ratios on growth performance, Ca and P balance, and bone mineralization in nursery pigs fed diets supplemented with phytase

**DOI:** 10.1093/tas/txad006

**Published:** 2023-01-09

**Authors:** Hengxiao Zhai, Jon Bergstrom, Jingcheng Zhang, Wei Dong, Zhenzhen Wang, Kostas Stamatopoulos, Aaron J Cowieson

**Affiliations:** DSM (China) Animal Nutrition Research Center, Bazhou 065799, China; DSM Nutritional Products, Parsippany, NJ 07054, USA; DSM (China) Animal Nutrition Research Center, Bazhou 065799, China; DSM (China) Animal Nutrition Research Center, Bazhou 065799, China; DSM (China) Animal Nutrition Research Center, Bazhou 065799, China; DSM Nutritional Products, Wurmisweg 576, 4303 Kaiseraugst, Switzerland; DSM Nutritional Products, Wurmisweg 576, 4303 Kaiseraugst, Switzerland

**Keywords:** bone, calcium, digestibility, phosphorus, phytase

## Abstract

The objective of this study was to investigate the effects of increasing dietary total Ca/total P ratios on growth performance, digestibility of Ca and P, bone mineralization, and concentrations of Ca and P in urine and plasma in nursery pigs. There were six diets in a randomized complete block design, including one positive control and five diets corresponding to five total Ca/total P ratios: 0.55, 0.73, 0.90, 1.07, and 1.24 (analyzed as 0.58, 0.75, 0.93, 1.11, and 1.30). These five diets were deficient in P but supplemented with 1,000 phytase units/kg feed. Each diet was fed to six pens of eight pigs (four barrows and four gilts per pen). All diets contained 3 g/kg TiO_2_, and fecal samples were collected from each pen on days 5–7 of trial. At the end, one pig per pen was sacrificed to collect the right tibia and urine in the bladder. The results showed that increasing dietary Ca/P ratio to 0.93 increased gain:feed but then gain:feed decreased as the Ca/P ratio was increased to 1.30 (linear and quadratic, *P* < 0.05). Although average daily gain and final BW were unaffected by changing Ca/P ratio in diet, dry bone weight; weights of bone ash, Ca and P; and bone Ca/P ratio increased linearly (*P* < 0.001) with increasing dietary Ca/P ratio. The percent bone Ca showed a tendency to increase (*P* = 0.064). Increasing dietary Ca/P ratio decreased apparent total tract digestibility (ATTD) of Ca and P linearly (*P* < 0.05) and the concentration of digestible P linearly (*P* < 0.001), but increased the concentration of digestible Ca (linear and quadratic effects: *P* < 0.01) and the digestible Ca/P ratio (linear effect: *P* < 0.001). In plasma, the concentration of Ca increased both linearly (*P* < 0.01) and quadratically (*P* = 0.051), whereas the concentration of P tended (linear and quadratic, *P* < 0.10) to decrease with increasing dietary Ca/P ratio. Similarly, in urine, the concentration of Ca increased both linearly and quadratically (*P* < 0.05), whereas the concentration of P decreased linearly (*P* < 0.01). In conclusion, increasing the dietary Ca/P ratio reduced feed efficiency but increased bone mass and the amounts of Ca and P deposited in bone of nursery pigs fed diets supplemented with 1,000 FYT/kg phytase. The increases in bone growth led to a reduction of urinary P excretion that exceeded the decreased digestible P supplied in diet with the widening dietary Ca/P ratios.

## Teaser

Pigs increase the efficiency of using digestible P for whole-body P retention by reducing urinary P excretion when fed diets with wider Ca/P ratios.

## INTRODUCTION

Adverse effects of increasing Ca/P ratios in diets without phytase on growth performance have previously been reported (Reinhart and Mahan, 1986; González-Vega et al., 2016a, 2016b; Lagos et al., 2019). The reduction in P digestibility due to the possible formation of insoluble Ca–P complexes in the gastrointestinal tract was considered one of the reasons (Stein et al., 2011; González-Vega et al., 2016a, 2016b). This agrees with the evidence that adverse effects of increasing dietary concentration of Ca on pig’s growth performance are more likely to be observed when diet P supply is deficient (Reinhart and Mahan, 1986; Wu et al., 2018) and these negative effects are ameliorated if P supply is above the requirement (González-Vega et al., 2016b; Lagos et al., 2019). The response of bone mineralization to increasing dietary Ca/P ratios appears to depend on whether the reduction in urinary excretion could compensate for the increase in fecal P output. The results of González-Vega et al. (2016b) showed that, when P supply was over the requirement, the reduction in urinary P excretion with increasing dietary Ca/P ratios could sustain an increase in P retention in bone and the whole body, whereas the reduction in urinary P excretion was negligible when P supply was deficient and thus a decrease in P retention occurs.

In diets supplemented with phytase, adverse effects of increasing dietary Ca/P ratios on both growth performance and bone mineralization have dominated the literature. In nursery pigs, Becker et al. (2020) observed a linear decrease in growth performance and bone mineral content when the Ca/available P ratio increased from 1.25 to 2.75 in diets supplemented with 250 U/kg phytase. Qian et al. (1996) reported adverse effects of increasing total Ca/total P ratio from 1.2 to 2.0 on growth performance, bone characteristics, and P digestibility in weanling pigs fed diets supplemented with 700 or 1,050 U phytase/kg feed. In growing–finishing pigs fed diets supplemented with 500 phytase U/kg, lowering the dietary total Ca/total P ratio from 1.5:1 to 1.3:1 to 1.0:1 improved growth performance and bone mineralization (Liu et al., 1998). The adverse effects reported in association with wider Ca/P ratios could be due to a reduction in phytase efficacy (Qian et al., 1996), the formation of insoluble phytate complex that is not accessible for hydrolysis by phytase (Selle et al., 2009), and/or overestimation of P release by phytase (Olsen et al., 2021). Most of these studies did not measure the digestibility of Ca and P from the diet along with their excretion through urine, which is helpful for gaining an improved understanding of the interactions among dietary Ca, P, and phytase and the growth performance and bone mineralization responses.

The aim of this study, therefore, was to investigate the effects of increasing dietary total Ca/total P ratios on the growth performance, digestibility of Ca and P, bone mineralization, and concentrations of Ca and P in urine and plasma in nursery pigs fed diets supplemented with 1,000 phytase unit (FYT)/kg feed. We hypothesized that bone mineralization will not be impaired when the dietary Ca/P ratio widens if the reduction in urinary excretion can compensate for the increase in fecal P output.

## MATERIALS AND METHODS

This study was conducted at DSM (China) Animal Nutrition Research Center Co. Ltd. (Bazhou, P. R. China) with its protocol approved by the Animal Welfare Committee of DSM (China) Animal Nutrition Research Center (AWCCAN).

### Animals and Facilities

In total, 288 barrows and gilts [PIC L1050 × L337; initial body weight (BW) 8.3 ± 0.7 kg (mean ± standard deviation)] were used in a randomized complete block design. The pigs were weaned at approximately 21 d of age and transferred to a nursery facility for an adaptation period of 7 d. In the nursery facility, each pen (space/pen = 3.0 × 1.8 m^2^) had a plastic-coated wire floor and was equipped with two water nipples and one stainless-steel feeder. Prior to the trial, the pigs were individually weighed and allotted into 36 pens based on their initial BW and gender (four barrows and four gilts per pen). The pens in each BW block were randomly assigned to the dietary treatments, resulting in six replicate pens per dietary treatment. The experimental diets were fed for 21 d. Feed and water were supplied *ad libitum*. At the end of trial, the pigs weighed 19.8 ± 1.9 kg.

Room temperature and ventilation were controlled by a computer system to provide an optimal environment. The room temperature was 27 °C at the start and gradually reduced to 23 °C at the end. The relative humidity was recorded to range from 40% to 70%.

### Experimental Diets

The ingredient and nutrient composition of the positive control and basal diets are presented in Table 1. The positive control diet was adequate in energy and nutrients, whereas the basal diet was deficient in Ca and P but supplemented with 1,000 FYT phytase/kg feed (HiPhorius, DSM Nutritional Products, Switzerland). The phytase was encoded by a 6-phytase gene from *Citrobacter braakii* and expressed in a strain of *Aspergillus oryzae*. The rice hulls in the basal diet were replaced by 0.05%, 0.25%, 0.45%, 0.65%, or 0.85% limestone to establish five experimental diets corresponding to total Ca/total P ratios of 0.55, 0.73, 0.90, 1.07, and 1.24. Titanium dioxide was included at 3 g/kg feed as an indigestible marker to enable the measurement of apparent total tract digestibility (ATTD) of Ca and P in all diets. All the diets were pelleted with a conditioning temperature at 75°C.

**Table 1.** Ingredient and nutrient composition of the positive control and basal diets (g/kg of feed, as-is basis)

Items	Positive control	Basal diet
Ingredients		
Corn	590.9	590.9
Soybean meal	340.5	340.5
Soybean oil	25.0	25.0
NaCl	4.5	4.5
NaHCO_3_	1.5	1.5
L-Lys·HCl	4.5	4.5
DL-Met	1.2	1.2
L-Thr	1.5	1.5
L-Val	1.0	1.0
Limestone	8.9	0.0
Monocalcium phosphate	9.5	2.5
Vitamin-mineral premix^1^	5.0	5.0
Rice hulls^2^	0.0	15.8
Benzoic acid	3.0	3.0
Phytase	0.0	0.1
TiO_2_	3.0	3.0
Total	1,000.0	1,000.0
Calculated nutrients and energy^3^		
Net energy, kcal/kg	2,499	2,499
Metabolizable energy, kcal/kg	3,372	3,372
Crude protein	218	218
Total Ca	6.2	2.1
Total P	5.6	4.1
Phytate P	2.5	2.5
Standardized total tract digestible P	3.4	2.0
Standardized ileal digestible		
Lys	12.9	12.9
Met	3.9	3.9
Thr	7.7	7.7
Trp	2.1	2.1
Val	8.3	8.3

^1^Premix supplied per kilogram of diet: vitamin A, 9,750 IU; vitamin D_3_, 3,000 IU; vitamin E, 63 mg; vitamin K_3_, 3.0 mg; vitamin B_1_, 3.0 mg; vitamin B_2_, 9.6 mg; vitamin B_6_, 4.5 mg; vitamin B_12_, 36 μg; D-biotin, 240 ug; D-calcium pantothenate, 30 mg; folic acid, 1.8 mg; niacin, 36 mg; Cu (tribasic copper chloride), 190 mg; I (potassium iodate), 0.6 mg; Fe (ferrous sulfate), 120 mg; Mn (manganese sulfate), 60 mg; Zn (zinc sulfate), 120 mg; Se (sodium selenite), 450 μg; choline (choline chloride), 300 mg; and Ca (calcium carbonate) 0.6 g.

^2^Rice hulls were replaced by limestone at 0.5, 2.5, 4.5, 6.5, and 8.5 g/kg feed to establish total Ca/total P ratios of 0.55, 0.73, 0.90, 1.07, and 1.24, respectively; rice hulls were analyzed to contain 0.09% Ca, 0.01% P, and 3.7% crude protein.

^3^Nutrients and energy were calculated without considering the contribution of rice hulls.

### Measurement and Sampling

The pigs were individually weighed on d 0 and 21 of trial, and the feed consumption per pen was recorded during the trial to calculate average daily gain, average daily feed intake and gain:feed.

Fresh and clean fecal samples were grabbed from each pen on d 5 to 7 of trial. Existing feces in each pen were removed before collection on each collection day. A total of approximately 600 g of fresh feces was collected per day per pen. All the fecal samples collected from each pen during the 3-d collection period were pooled and mixed to homogeneity with a hand-held blade mixer (TD-110, RuiBao Hardware Co. Ltd., Dongguan, P. R. China). A sub-sample of approximately 500 g for each pen was stored at −20 °C until further processing.

Blood, the right tibia, and urine from the bladder were collected from the pig in each pen with BW closest to the average BW per pen on d 21 of trial. Blood samples were collected from the vena cava into heparin vacutainer collection tubes and immediately centrifuged at 3,000 × g for 10 min to obtain plasma. All the samples were stored at −20 °C before processing. The tibias were processed according to the nondefatting bone-processing procedures described by Wensley et al. (2020). In short, the bones were autoclaved at 120 °C for 30 min to facilitate the removal of muscular tissues and cartilaginous caps. The cleaned bones were left at room temperature for 1 d and then oven-dried at 105 °C for 7 d. Finally, the dried tibias were incinerated in a muffle oven for 72 h at 600 °C.

### Chemical Analyses

The fecal samples were oven-dried to a constant weight and ground to pass through a 0.5-mm screen before analysis. The dietary and fecal samples were dried at 105 °C in an oven for 4 h for dry matter determination (method 934.01; AOAC International, 2006). Titanium, Ca, and P were determined by Inductively Coupled Plasma–Optical Emission Spectrometry (ICP–OES; Optima TM 8000, PerkinElmer, Shelton, USA; method 985.01; AOAC International, 2006) after microwave digestion. Urine samples (10 mL) were dried at 60 °C before the microwave digestion. Plasma Ca and P were analyzed on a chemistry analyzer (AU480, Beckman Coulter, Brea, USA). The phytase activity was determined by colorimetric measurement of the released phosphate from phytate. One phytase unit was defined as the amount of enzyme that releases 1 µmol of inorganic phosphate from 50 mM phytate per min at 37 °C and pH 5.5. These analyses were performed in duplicate, except that phytase activity in the feed samples was determined from three replicates.

### Calculations and Statistical Analyses

The experiment was a randomized complete block design. Each pen or pig was an experimental unit.

Digestibility of Ca and P were calculated using the following equation:


$$\begin{array}{l} D\;=\;[1\;-\;\left(T_{i}/T_{o}\right)\;\times \;\left(M_{o}/M_{i}\right)]\;\;\times \;\;\;100; \end{array}$$

where D is the ATTD of Ca or P (%); T_i_ and T_o_ are the titanium concentrations in diet and feces, respectively (% of DM); M_i_ and M_o_ are the concentrations of Ca or P in diet and feces (% of DM), respectively. The digestible Ca and P were calculated by multiplying the concentrations of Ca and P in feed (g/kg feed) by their corresponding ATTD coefficients. The P retention was calculated by deducting the urinary P excretion from the digestible P. The urinary P excretion was estimated by multiplying the urinary P concentration with an assumed urine output of 2 liters per kg feed intake. It should be noted that the urinary P measurement was performed 2 wk after the fecal collection.

The results were analyzed using the MIXED procedure of SAS 9.4 (SAS Inst. Inc., Cary, NC) with the model including the dietary treatment as a fixed effect, replicate as a random effect and an error term. Polynomial orthogonal contrasts were constructed to test the linear and quadratic effects of Ca/P ratios and to compare the PC and the other diets. The linear and quadratic regression equations were constructed with Microsoft Excel 2202 (Microsoft Corp., Redmond, WA). The least square means were presented, and the significance was defined at *P* < 0.05.

## RESULTS

### Experimental Diets and the Analyses

There was good agreement between the formulated and analyzed concentrations of Ca and P in the experimental diets (Table 2). The analyzed Ca in phytase diets increased from 0.24% to 0.52% with increasing inclusion rate of limestone, which agrees well with the target range of 0.23% to 0.52%. Accordingly, the analyzed Ca/P ratio was increased from 0.58 to 1.30, which was slightly higher than the target ratio range of 0.55 to 1.24, and thus the actual Ca/P ratios were used for the contrast analysis. The analyzed phytase activities were within ± 15% of the intended targets.

**Table 2.** Formulated and analyzed calcium and phosphorus content (as-is basis, %) and phytase activity (FYT/kg) of dietary treatments

	Positive control	Dietary Ca/P ratio				
		0.55	0.73	0.90	1.07	1.24
Formulated						
Ca	0.62	0.23	0.30	0.37	0.44	0.52
P	0.56	0.41	0.41	0.41	0.41	0.41
Ca/P	1.10	0.55	0.73	0.90	1.07	1.24
Phytase	0	1,000	1,000	1,000	1,000	1,000
Analyzed						
Ca	0.63	0.24	0.31	0.38	0.45	0.52
P	0.58	0.42	0.42	0.41	0.41	0.40
Ca/P	1.09	0.58	0.75	0.93	1.11	1.30
Phytase	0	1,083	1,077	1,033	1,121	1,101

### Growth Performance and Bone Mineralization

**Figure 1.**
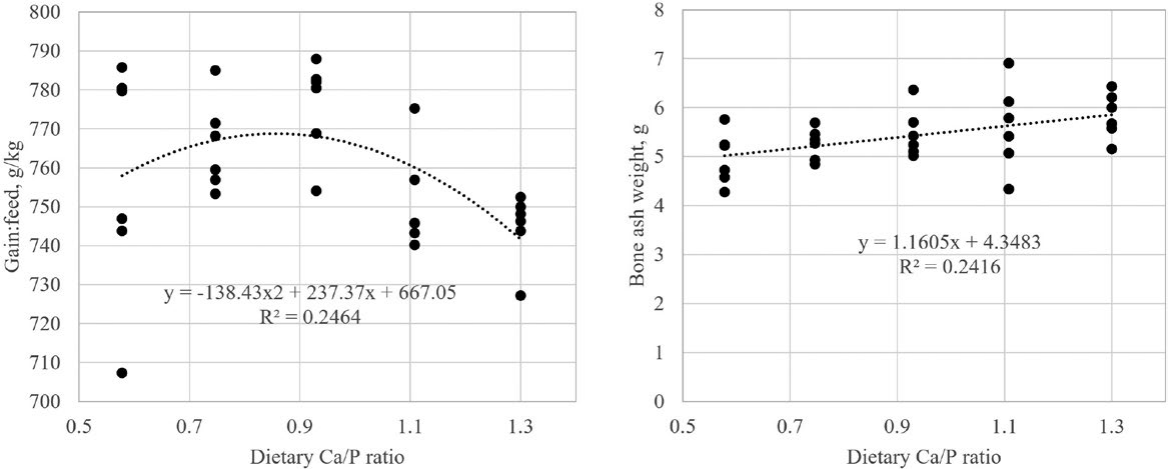
Regression of gain:feed and bone ash weight against analyzed dietary Ca/P ratios.

Increasing the dietary Ca/P ratio to 0.93 increased gain:feed but then gain:feed decreased as the Ca/P ratio was increased to 1.30 (linear and quadratic, *P* < 0.05; Table 3). By fitting a quadratic model, the maximal gain:feed was calculated to occur at an approximate Ca/P ratio of 0.86 (Figure 1).

**Table 3.** Growth performance of the pigs fed diets with different Ca/P ratios^1^

Item^2^	Positive control	Dietary Ca/P ratio					SEM	PC vs. others	Dietary Ca/P ratio	
		0.55	0.73	0.90	1.07	1.24			L^4^	Q^4^
IBW, kg	8.3	8.3	8.3	8.3	8.3	8.3	0.01	0.756	0.823	0.531
FBW, kg	19.7	19.8	19.9	20.2	19.8	19.6	0.22	0.624	0.528	0.123
ADG, g	546	548	553	570	548	540	10.2	0.617	0.531	0.107
ADFI, g	720	724	723	735	729	726	14.0	0.659	0.836	0.666
Gain:feed, g/kg	758	758	766	776	751	744	5.7	0.920	0.029	0.006

^1^There were six replicate pens.

^2^IBW, initial body weight; FBW, final body weight; ADG, average daily gain; ADFI, average daily feed intake; SEM: standard error of the mean.

^3^Linear and quadratic effects of dietary Ca/P ratio.

Increasing dietary Ca/P ratio increased dry bone weight; weights of bone ash (Figure 1), Ca and P; and bone Ca/P ratio linearly (*P* < 0.001; Table 4). The percent bone Ca also tended to increase linearly (*P* = 0.064). The pigs fed PC diet had heavier weights of dry bone, bone ash and bone Ca and P, and a higher bone Ca/P ratio than the average of pigs fed the phytase diets (*P* < 0.05).

**Table 4.** Bone mineralization of the pigs fed diets with different Ca/P ratios^1^

	Positive control	Dietary Ca/P ratio					SEM^2^	PC *vs* others	Dietary Ca/P ratio	
		0.55	0.73	0.90	1.07	1.24			L^3^	Q^3^
Bone, g	12.5	10.6	11.2	11.7	11.9	12.0	0.29	0.003	< 0.001	0.223
Bone ash, %	48.5	47.0	47.1	46.9	47.1	48.8	1.01	0.315	0.256	0.361
Bone Ca, %	17.6	16.6	16.6	16.8	17.0	17.7	0.39	0.155	0.064	0.385
Bone P, %	8.9	8.7	8.6	8.6	8.7	8.9	0.20	0.369	0.393	0.444
Bone ash, g	6.1	5.0	5.3	5.5	5.6	5.8	0.17	0.002	< 0.001	0.719
Bone Ca, g	2.2	1.8	1.9	2.0	2.0	2.1	0.06	< 0.001	< 0.001	0.756
Bone P, g	1.1	0.9	1.0	1.0	1.0	1.1	0.03	0.003	0.001	0.670
Bone Ca/P	1.98	1.92	1.94	1.95	1.96	1.99	0.011	0.023	< 0.001	0.656

^1^There were six replicate pigs.

^2^SEM: standard error of the mean.

^3^Linear and quadratic effects of dietary Ca/P ratio.

### Digestibility of Ca and P, and Digestible Ca and P, in Experimental Diets

Increasing dietary Ca/P ratio linearly (*P* < 0.05) decreased ATTD of Ca and P, and the ATTD of Ca also tended (*P* < 0.10) to be quadratic (Table 5). With increasing dietary Ca/P ratio, the concentration of digestible Ca in diet increased both linearly and quadratically (*P* < 0.01), the digestible Ca/P ratio increased linearly (*P* < 0.001), but the concentration of digestible P decreased linearly (*P* < 0.001).

**Table 5.** Apparent total tract digestibility (ATTD) of Ca and P in experimental diets with different Ca/P ratios^1^

	Positive control	Dietary Ca/P ratio					SEM^2^	PC *vs* others	Dietary Ca/P ratio	
		0.55	0.73	0.90	1.07	1.24			L^3^	Q^3^
ATTD of Ca, %	57.1	76.7	75.8	78.5	74.5	73.5	1.0	< 0.001	0.017	0.076
ATTD of P, %	53.4	73.9	72.3	73.6	70.7	69.4	0.8	< 0.001	< 0.001	0.253
Digestible Ca, g/kg	3.62	1.85	2.35	3.02	3.37	3.86	0.04	< 0.001	< 0.001	0.009
Digestible P, g/kg	3.09	3.08	3.00	3.04	2.89	2.80	0.04	0.004	< 0.001	0.152
Digestible Ca/P	1.17	0.60	0.78	0.99	1.16	1.38	0.01	< 0.001	< 0.001	0.604

^1^There were six replicate pens.

^2^SEM: standard error of the mean.

^3^Linear and quadratic effects of dietary Ca/P ratio.

In comparison to the PC diet, the phytase diets on average gave higher ATTD of Ca and P, but less ingested digestible Ca and P per kg of diet with lower digestible Ca/P ratio (*P* < 0.01).

### Concentrations of Ca and P in Plasma and Urine

In plasma, the concentration of Ca increased both linearly (*P* < 0.01) and quadratically (*P* = 0.051), whereas concentration of P tended to decrease (linear and quadratic, *P* < 0.10) with increasing dietary Ca/P ratio (Table 6). In urine, the concentration of Ca increased both linearly and quadratically (*P* < 0.05), whereas the concentration of P decreased linearly (*P* < 0.01) with increasing dietary Ca/P ratio. The pigs fed the PC diet tended (*P* < 0.10) to have an increased concentration of Ca in plasma but had significantly greater (*P* < 0.05) Ca in urine when compared to the mean of the phytase treatments.

**Table 6.** Concentrations of calcium and phosphorus in plasma and urine of pigs fed diets with different Ca/P ratios^1^

	Positive control	Dietary Ca/P ratio					SEM^2^	PC vs. others	Dietary Ca/P ratio	
		0.55	0.73	0.90	1.07	1.24			L^3^	Q^3^
Plasma, mg/L										
Ca	113	93	109	109	110	112	4	0.096	0.002	0.051
P	87	97	93	99	99	78	6	0.319	0.098	0.080
Urine, mg/L										
Ca	217	4	4	4	17	278	59	0.038	0.012	0.036
P	44	533	304	250	38	8	122	0.224	0.006	0.593

^1^There were six replicate pigs.

^2^SEM: standard error of the mean.

^3^Linear and quadratic effects of dietary Ca/P ratio.

## DISCUSSION

Detrimental effects of increasing dietary Ca/P ratio on the growth performance and bone mineralization of pigs have previously been reported. For example, Qian et al. (1996) reported harmful effects of increasing Ca/P ratio from 1.2 to 2.0 on growth performance and bone characteristics in weanling pigs fed diets supplemented with phytase. More recently in nursery pigs, a linear decrease in growth performance and bone mineral content was observed when the Ca/available P ratio increased from 1.25 to 2.75 in diets with phytase (Becker et al., 2020). These adverse effects on pig growth were more likely to occur when the dietary P supply was deficient (Wu et al., 2018), and a reduction of digestible P in diet with increasing Ca may have exacerbated the deficiency (González-Vega et al., 2016a). Indeed, we observed a linear reduction in both the digestibility of P and the concentration of digestible P in diet with increasing Ca/P ratio, which coincided with the deteriorating feed efficiency when the Ca/P ratio exceeded either 0.93 or 1.11 in the current study. Moreover, soft tissue development is mainly dependent on P even though both Ca and P are essential (Lautrou et al., 2020), and there was a close relationship between whole-body P mass and empty BW (Bikker and Bloc, 2017). The reduction in digestible P from 2.2 to 2.0 g/d with increasing Ca/P ratio in the current study could indicate that the dietary P supply had become marginally deficient when considering the average requirement of 2.16 g/d for 7 to 11 and 11 to 25 kg pigs (NRC, 2012). Reduced P digestibility can be caused by an impairment in phytase efficacy (Qian et al., 1996), poor accessibility of Ca–phytate complexes by phytase (Selle et al., 2009), and the formation of insoluble Ca–P complexes in the gastrointestinal tract (Stein et al., 2011). However, a reduction in digestibility of P can still accompany an increase in whole body P retention (González-Vega et al., 2016b); and, therefore, we believe that other potential reasons behind poor growth performance with wider Ca/P ratios are worth exploring. Johnston et al. (2004) found that the reduction in dietary Ca and P was just as effective as phytase addition in increasing the digestibility of nutrients and thus some negative impact on nutrient availability could be expected with increasing dietary Ca/P ratio. The antinutritional effects of residual phytic acid could be augmented due to its recalcitrancy to phytase in the presence of extra Ca. After all, phytic acid has been shown to negatively impact amino acids availability (Pontoppidan et al., 2007), starch and protein digestion (Thompson et al., 1987; Yu et al., 2012), fat utilization (Govers and Van der Meer, 1993), and endogenous losses of amino acids and minerals (Cowieson et al., 2009). In addition, the possible formation of Ca–phytate complexes implies there could be less liberation and absorption of *myo*-inositol for potential extra-phosphoric effects relating to protein accretion (Schmeisser et al., 2017; Lu et al., 2019).

Bone mineralization requires both Ca and P. A deficient P supply when Ca supply was adequate reduced absorption and retention of P, as well as Ca, and ultimately led to poor bone mineralization (Sørensen et al., 2018, 2019). A deficient Ca supply when P supply was adequate reduced bone formation and mineralization rates, which were partly due to the secondary hyperparathyroidism that the pigs developed to maintain the plasma Ca concentration (Eklou-Kalonji et al, 1999). In contrast, an excessive supply of P did not result in additional bone mineral retention in pigs (Sørensen, et al., 2018, 2019), whereas an excessive supply of Ca could reduce P digestibility and impair growth performance and skeletal development (Stein et al., 2011; Becker et al., 2020). The current study provides an example where digestible Ca supply increased whilst digestible P supply decreased. Specifically, the amount of digestible Ca in diet increased with increasing Ca/P ratio in contrast to the decrease in ATTD of Ca; however, both the amount of digestible P and ATTD of P decreased. Increasing dietary Ca will increase the amount of retained P as long as Ca and P are balanced (Létourneau-Montminy et al., 2012). Therefore, the increase in the absorbed Ca was likely responsible for the improvement in bone development with increasing dietary Ca/P ratio and might have constrained the amount of P available for protein deposition in the current study. The improved bone mineralization in this study was manifested with an increase in bone mass and the amounts of Ca, P, and ash in the bone, but the proportion of these components did not change significantly except for the tendency for the percentage of Ca in bone to increase. It appeared that the growth in bone mass was predominant under the current experimental conditions, which is supported by the finding of Lagos et al. (2019) that bone tissue synthesis is primarily manifested by the change of bone size, whereas the bone ash percentage remains constant. In the same vein, weights of ash, Ca and P in bone were found to better represent bone mineralization than their percentages (Lee et al., 2021). Percent bone ash is more affected by dietary P supply than Ca/P ratio (Reinhart and Mahan, 1986). The Ca/P ratio in bone, however, increased with higher Ca/P ratios in the diet, which agrees with our previous study (Zhai et al., 2022) and indicates certain plasticity in bone mineral composition.

**Figure 2.**
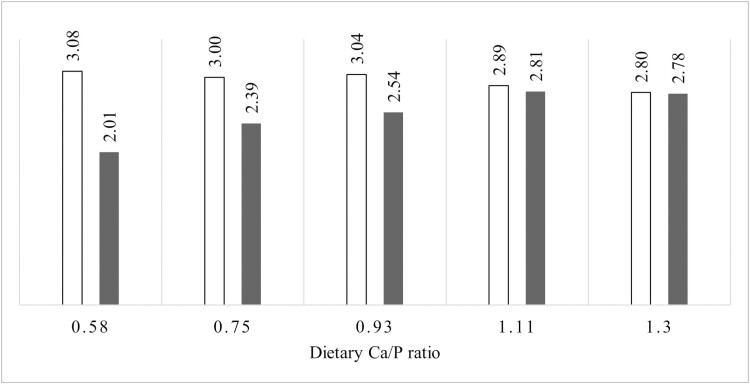
Comparison of apparent digestible P (g/kg feed, open bars) and P retention (g/kg feed, solid bars) in experimental diets for each of the analyzed Ca to P ratios. The P retention was estimated by assuming that 2 L of urine was voided for each kg of feed intake.

Ca absorption is a function of the concentration of free, ionized Ca in the intestinal lumen, and the effects of dietary factors such as phytate can be largely explained by their interaction with this free cation (Allen, 1982). In diets not supplemented with phytase, the ATTD of Ca was not significantly affected by the dietary concentration of Ca (Stein et al., 2011; González-Vega et al., 2016b). We observed higher ATTD of Ca in the phytase-supplemented diets than in the PC, which could be linked with the breakdown of phytate due to the supplementation of phytase and the associated liberation of Ca bound by phytate and inhibition of phytate binding to free Ca. The increase in concentration of digestible Ca in diet with higher dietary Ca/P ratios led to an increase in the concentration of Ca both in plasma and urine but, on the contrary, the concentration of P showed a tendency to decrease in plasma and a significant decrease in urine. This contrast implies that the pigs strived to maintain a systemic Ca/P balance by minimizing the urinary excretion of P and increasing the urinary excretion of Ca. If we assumed that 2 liters of urine was voided for 1 kg of feed intake for pigs in the current study based on the observation that urine output ranged from 2.7 to 4.3 L/kg feed intake in 23 kg pigs (Stein et al., 2011), the reduction in urinary P excretion equated to a gain of 1.0 g digestible P per kg feed as the dietary Ca/P ratio increased from 0.58 to 1.30, which was greater than the decrease in digestible P of 0.28 g/kg feed. Such an improvement in the efficiency of using digestible P for whole-body retention of P likely explains the observed improvement in the amounts of Ca and P deposited in bone with increasing dietary Ca/P ratio despite the declining supply of digestible P in diet as illustrated in Figure 2. In other words, the pigs retained more P by reducing the urinary excretion of P. Similarly, González-Vega et al. (2016b) observed that urinary P output decreased by 0.46 g/d in contrast to an increase of 0.26 g/d in fecal P output when the STTD Ca increased from 0.32% to 0.72%, which resulted in a net increase in P retention. This compensatory mechanism has its limit, however, and depends on the adequacy of dietary P supply. In the studies by Sørensen et al. (2018, 2019), deficient P supply when Ca supply was adequate reduced retention of P and bone mineralization despite the pig’s adaptation to reduce urinary excretion of P and increase urinary excretion of Ca.

The effect of dietary Ca/P ratio on the efficacy of phytase in terms of P release could not be judged in the current study due to lack of control diets without phytase, and thus attributing the observed decrease in digestible P with increasing dietary Ca/P ratio to a reduction in phytase efficacy would be arbitrary. The above-mentioned adverse effects of widening dietary Ca/P ratio on growth performance, bone mineralization, and P digestibility were often attributed to a reduction in phytase efficacy. Liu et al. (1998) listed three potential mechanisms: 1) the formation of an insoluble phytate complex, 2) the increase in the pH of the intestinal digesta, and 3) the direct suppression on phytase activity. In diets without phytase supplementation, however, the decrease in digestibility of P with increasing concentration of dietary Ca has been well recognized (Stein et al., 2011). Moreover, increasing the dietary Ca/P ratio from 1.3 to 1.9 reduced P digestibility equally in diets with and without phytase and thus did not influence the efficiency of phytase in terms of releasing P (Létourneau-Montminy et al., 2010). More research is warranted to clarify whether the negative effect of ingesting high levels of Ca on P digestibility is direct or via interactions with phytase or phytate. To precisely integrate this negative impact on P digestibility in connection with wider Ca/P ratios in diet formulation is still a challenge. Assigning lower P matrix values to phytase in reflection of this negative impact might be a practical approach for nutritionists.

## CONCLUSION

Increasing the dietary Ca/P ratio above 0.86 reduced feed efficiency, but increased bone mass and the amounts of Ca and P deposited in bone of nursery pigs fed diets supplemented with 1,000 FYT phytase/kg feed. The impairment in feed efficiency could have been caused by other factors besides the simple reduction in digestible P supply resulting from wider dietary Ca/P ratios. The increases in bone growth appeared to be supported by the conservation of P with a reduced excretion through urine that exceeded the reduction in digestible P supply from the diet.
